# Self-similarity of low-frequency earthquakes

**DOI:** 10.1038/s41598-020-63584-6

**Published:** 2020-04-16

**Authors:** M. Supino, N. Poiata, G. Festa, J. P. Vilotte, C. Satriano, K. Obara

**Affiliations:** 1 Université de Paris, Institut de physique du globe de Paris, CNRS, F-75005 Paris, France; 2 Dipartimento di Fisica ‘Ettore Pancini’, Università di Napoli Federico II, I-80126 Napoli, Italy; 3 National Institute for Earth Physics, 12 Călugăreni, Măgurele, 077125 Ilfov Romania; 4 Earthquake Research Institute, University of Tokyo, Bunkyo, Tokyo 113-0032 Japan

**Keywords:** Geophysics, Seismology, Tectonics

## Abstract

Low-frequency earthquakes are a particular class of slow earthquakes that provide a unique source of information on the physical processes along a subduction zone during the preparation of large earthquakes. Despite increasing detection of these events in recent years, their source mechanisms are still poorly characterised, and the relation between their magnitude and size remains controversial. Here, we present the source characterisation of more than 10,000 low-frequency earthquakes that occurred during tremor sequences in 2012–2016 along the Nankai subduction zone in western Shikoku, Japan. We show that the scaling of seismic moment versus corner frequency for these events is compatible with an inverse of the cube law, as widely observed for regular earthquakes. Their radiation, however, appears depleted in high-frequency content when compared to regular earthquakes. The displacement spectrum decays beyond the corner frequency with an omega-cube power law. Our result is consistent with shear rupture as the source mechanism for low-frequency earthquakes, and suggests a self-similar rupture process and constant stress drop. When investigating the dependence of the stress drop value on the rupture speed, we found that low-frequency earthquakes might propagate at lower rupture velocity than regular earthquakes, releasing smaller stress drop.

## Introduction

Worldwide, seismic and geodetic observations recorded along a number of subduction zones^1–5^ and continental faults^6–8^ have revealed a broad class of transient energy-release signals known as slow earthquakes. Geodetic slow earthquakes^9–12^ are slow slip events (SSEs) with durations of days (short-term SSEs) or months to years (long-term SSEs). Seismic slow earthquakes are characterised by lower dominant frequencies than regular earthquakes of the same moment. These are impulsive low-frequency earthquakes (LFEs) and tectonic tremor signals with dominant frequencies in the 1–10 Hz band^13–16^, and very-low-frequency earthquake (VLFE) signals with dominant periods in the 10 s to 100 s band^17–20^.

Numerous observations have shown that tectonic tremors, LFEs, VLFEs and SSEs often accompany each other and occur in ductile-to-brittle environments at the neighbouring sides of large earthquake-producing seismogenic zones^21^. Recent observations have suggested that SSEs might trigger megathrust earthquakes^22,23^. As such, detailed characterisation of slow earthquakes activity might represent a unique source of information to improve seismic hazard monitoring and risk assessment^21^. It is often assumed that slow earthquakes provide sparse observations that probe different scales of a common transient process along slowly driven plate boundaries. While this physical process remains to be fully understood, a linear scaling between moment and source duration across the different slow earthquake observation scales has been proposed^24^ and interpreted as the signature of a different process to that for regular earthquakes, or alternatively as the signature of a scale-bound source process for the longest duration events^25^.

Low-frequency earthquakes are often observed in association with SSEs on the deep extensions of plate boundaries. They typically occur in burst-like sequences of a multitude of events mixed in with long-lasting tectonic tremor signals. In recent years, advanced data analysis methods have been developed to improve detection of LFEs^26–28^, and very large datasets are becoming available to the scientific community. However, the source mechanism and scaling properties of these events still remain poorly known; the main difficulty being the very low signal-to-noise ratio associated with these transients. Bostock *et al*.^29^ reported an almost constant source duration for ~100 LFE templates along the Cascadia plate boundary, albeit over a limited moment range. This result is in contrast with classical observations for regular, fast earthquakes^30^, where the seismic moment is proportional to the cube of the source duration.

## The Nankai subduction zone

**Figure 1:**
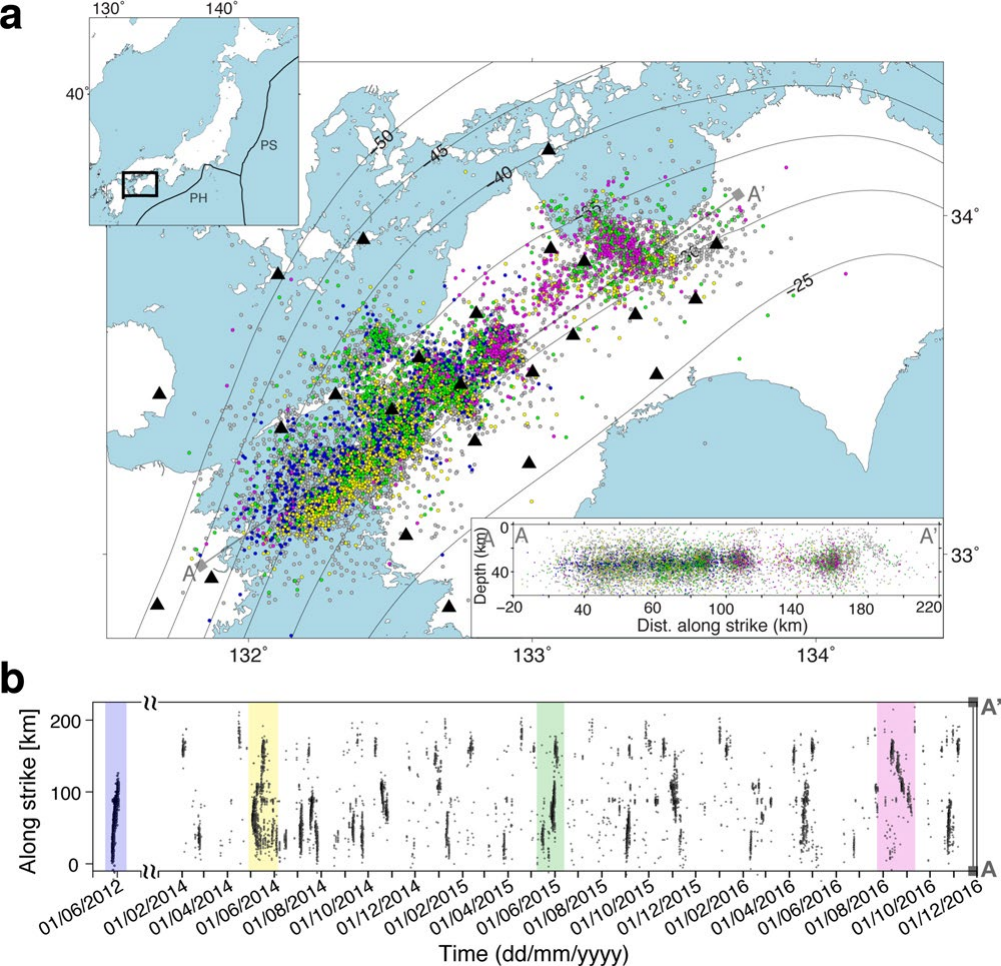
Distribution of low-frequency earthquakes. (**a**), Map of the locations of the analysed low-frequency earthquakes (circles) and the Hi-net stations (triangles). The events are coloured as follows: blue, yellow, green and magenta circles indicate low-frequency earthquakes extracted from the time-periods corresponding to the largest tectonic tremor sequence of each analysed year and occurring during this tremor sequence; grey circles correspond to all other events. Inset, top: The geographic location of the western Shikoku area and the main tectonic features, such as the geometry of the plate interface (grey contours) and the main tectonic plates (Philippine Sea: PS and Pacific: PA), for Japan. Inset, bottom: Depth cross-section of the analysed events projected along the strike of N 40° E (A-A’). (**b**), Space–time plot of the events. Coloured rectangles mark the time-periods of largest tremor sequences, corresponding to event coloured as in (a).

Here, we present the source characteristics of 10,157 LFEs extracted from seismic data recorded during the periods of intense tectonic tremor activity, or tectonic tremor sequences, that occurred during the periods of May to June 2012 and January 2014 to November 2016, along the Nankai subduction zone in western Shikoku, Japan (Fig. 1).

In this region, the Philippine Sea Plate is subducting beneath Japan, with a recurrence time of megathrust earthquakes from 100 to 150 years^31^. We analysed the velocity seismograms recorded at 25 stations of the high-sensitivity borehole seismic network (Hi-net), managed by the National Research Institute for Earth Science and Disaster Prevention (NIED), Japan^32,33^. The massive catalogue of LFEs (Fig. 1; Supplementary Fig. 1) has been obtained by exploiting the coherency of characteristic function of the wave field recorded across the network stations, using the automatic network-based method BackTackBB^28,34^ during the periods of significant tectonic tremor activity defined based on NIED tectonic tremor catalog^35,36^. The method exploits frequency-dependent higher-order statistical characteristics of the signal to extract and localise in time the onset of short-duration LFE transients within the continuous seismic signals, and uses their coherency across the seismic network to locate the LFE sources in space and time. The methodological processing and analysis steps, together with the set-up parameters, are detailed in Poiata *et al*.^34^.

## Source characterisation

We processed the data and characterised the source parameters for each of the events in the catalogue. We modelled the S-wave displacement amplitude spectrum of the LFEs using a generalised Brune’s spectral model^37^ (see Methods). We assumed a horizontally layered one-dimensional propagation model, and a constant frequency-independent anelastic attenuation factor *Q*, which are validated approximations for the investigated area^38^.

After removal of the Green’s propagator, the source spectrum is assumed to be flat at low frequencies and to decay as a power law at high frequencies, with a crossover region around a cut-off corner frequency *f*_*c*_. The parameters to be retrieved are: the flat spectrum level, which is proportional to the seismic moment and a proxy for the event magnitude; the corner frequency, which is related to the event size; and the power-exponent of the high-frequency fall-off, which constrains the energy radiated by the earthquake. The source parameters are estimated by inversion of the displacement spectra using a probabilistic approach^39^. This method evaluates the joint probability density functions (PDFs) of the source parameters allowing robust estimations that account for the correlations between parameters and the related uncertainties.

For each LFE, we inverted each individual station displacement spectrum. The marginal PDFs of each source parameter were retrieved integrating the joint PDF (see Supplementary Fig. 2; see Methods). Extremely low signal-to-noise-ratio observations were automatically detected and rejected (see Methods). The source parameters of a LFE recorded at more than one station were estimated as the weighted means of the single station solutions (see Methods), as seismic moments and corner frequencies inferred from different stations show some variability (Supplementary Fig. 3), as for regular earthquakes.

## Scaling of corner frequency with seismic moment

**Figure 2:**
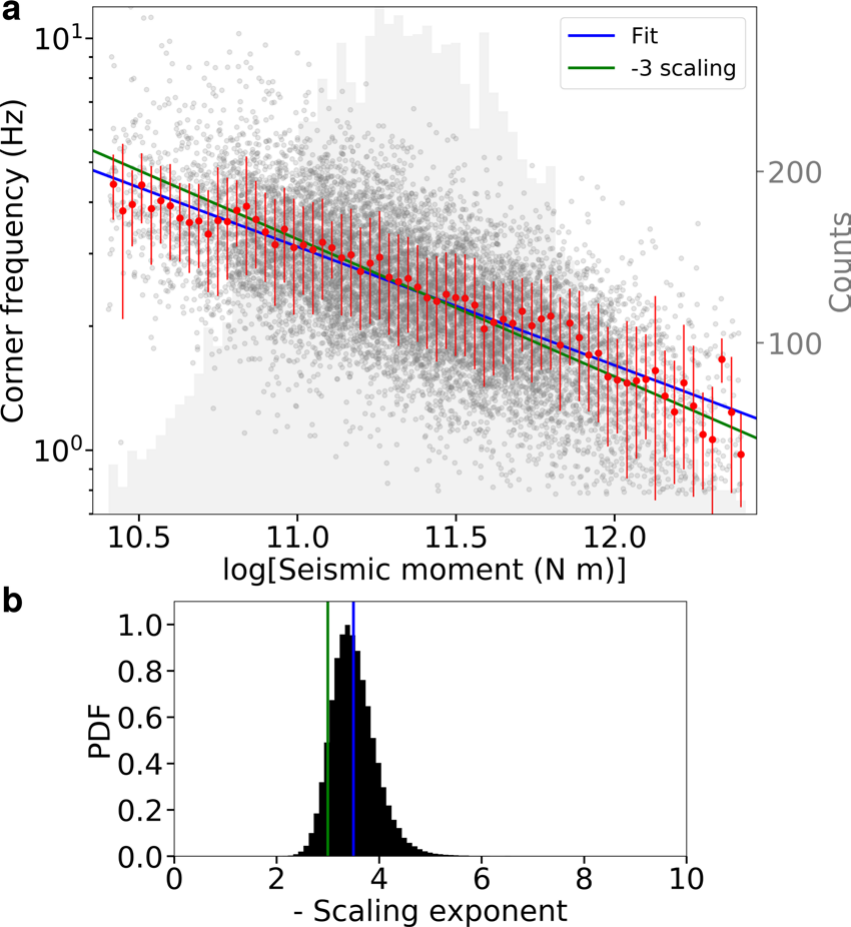
Scaling of the corner frequency with the seismic moment. (**a**), The corner frequency and the seismic moment estimates for each LFE are shown (grey points). The weighted averages of the corner frequencies for the selected seismic moment bins (bin-size, 0.03) are shown (red points), along with the weighted standard deviations per bin (red bars). The best-fit curve (blue line) of the averaged estimates (red points) has a scaling parameter of −3.5. The histogram in the background (grey shading) shows the number of events in each bin. The green line represents the scaling of −3. (**b**), Probability density function of the scaling parameter estimated by a bootstrap method performed with 100,000 random extractions (see Methods); colours as for the top panel.

The estimated corner frequencies and seismic moments of the analysed LFEs are shown in Fig. 2.

The source parameters are well resolved for the whole range of the seismic moments explored. As an example, we show the velocity records, the displacement spectra and the solutions for three events from the ends and the middle of the explored seismic moment range (Supplementary Figs. 2, 4, 5). For the same range, we also present three synthetic tests (Supplementary Fig. 6) where we inverted the spectrum obtained by adding to the theoretical source spectrum a noise spectrum which decreases with increasing frequency, as observed in real cases. The corresponding solutions are well constrained around the true value of the source parameters, showing that the inversion is reliable also for the low signal-to-noise ratio characterizing these events.

The LFEs source characteristics showed typical behaviours^40,41^, with corner frequencies much lower than expected for regular earthquakes of the same magnitude. The high-frequency fall-off exponents have a median of 3.0, with 80% of the events between 1.9 and 4.1 (Supplementary Fig. 7a).

The scaling between the corner frequency and the seismic moment is clear (Fig. 2). To deal with the large number of solutions, we grouped the corner frequency estimations into log *M*_0_ bins with a size of 0.03. The histogram representing the number of points per bin is shown as shaded background in Fig. 2a. For each bin, we computed the weighted average of the corner frequencies and the uncertainty related to the data variability (Fig. 2a, red points and error bars; see Methods). We assumed the uncertainty on the seismic moments as negligible since the associated relative uncertainty (1.2%) is on average more than one order of magnitude smaller than that of the corner frequencies (22%)^42^. Using this averaged information, we performed a linear regression according to Eq. (1):

1 $$\log \,{f}_{c}=A\,\log \,{M}_{0}+B$$

where *A* and *B* are constants to be determined (Supplementary Table 1). We obtained a scaling parameter ${A}^{\text{'}}\equiv 1/A=-3.5$. We used an unweighted regression that assigns the same weight to each bin and avoids the fit to be dominated by the central regions in the seismic moment domain. Nevertheless, when using weighted linear regression the estimated scaling parameter of ${A}^{\text{'}}=-3.35$ remains very close to the previous estimation.

The PDF of the scaling parameter is estimated by a Bootstrap method (Fig. 2b; see Methods) to assess the robustness of the result. We found that the scaling parameter is normally distributed with an expected value of $-3.5\pm 0.5$. Our result is consistent within a 1-sigma confidence interval with the classical −3 scaling parameter observed for regular earthquakes^30^.

When the seismic moment domain is reduced to only one decade (log *M*_0_ = 11.0–12.0), the mean estimated value of the scaling parameter is −3.5 with a mode of −3.2 (Supplementary Fig. 8a). For this test, only bins with at least 100 observations (Fig. 2a) are retained.

The effects of a constant anelastic attenuation factor on the *M*_0_ - *f*_*c*_ scaling was assessed by reprocessing the data with different constant attenuation factors *Q* = 100 and *Q* = 500, which are lower and higher, respectively, than the value provided in the literature (Q = 300)^38^. Results show a variation of about 15% ($-4.0\pm 0.6$) for Q = 100, and of about 3% ($-3.6\pm 0.5$) for Q = 500.

Moreover, we reprocessed the data with a frequency-dependent attenuation factor:

2 $${\rm{Q}}({\rm{f}})={{\rm{Q}}}_{0}{{\rm{f}}}^{{\rm{\varepsilon }}},$$

where log (Q_0_)^−1^ = −2.5 and ε = 0.5 as provided in literature^43^. Results (Supplementary Fig. 9) show a variation in the estimated power law exponent of about 6% ($-3.7\pm 0.5$). For these three tests, we retrieved a mean value of the exponent of the high-frequency spectral fall-off ranging between 3.1 and 3.2 with a median value of 2.9, indicating that the estimation of the high-frequency decay exponent is not actually affected by the specific selection of the quality factor (Supplementary Fig. 7).

We also addressed the possibility that a −3.5 scaling parameter might arise when LFE clusters from distinct tremor sequences with different scaling are collated. We thus estimated independently the scaling of different subsets of LFEs clustered in space and time (Supplementary Fig. 10a). For doing this, we selected the LFEs composing the largest tremor sequence of each analysed year (Fig. 1). Each cluster shows a similar scaling parameter to that derived from the entire catalogue, over almost the same seismic moment range (Supplementary Fig. 10b).

Finally, we evaluated the source parameters of 2315 LFEs extracted from the unified earthquake catalogue provided by the Japanese Meteorological Agency (JMA)^44,45^, for the same region and time period as those investigated in this study. The source location of LFEs in this catalogue is determined using manually picked arrivals of P- and S-waves. We found a scaling parameter of −3.0 (Supplementary Fig. 11a). We also retrieved an exponent with a median value of 2.8 for the high-frequency spectral fall-off (Supplementary Fig. 11b).

## Similar scaling law for LFE and regular earthquake sources

From the analysed LFEs, we retrieved a power-law scaling between seismic moment and corner frequency similar to that observed for regular earthquakes. The seismic moment scales as the inverse of the cube of the corner frequency (Fig. 2). This is consistent with a shear rupture process at the LFEs source. Similar results have been recently reported for the scaling of SSEs source in Cascadia^46^ and Mexico^47^, and for long-period events in volcanic environments^48^.

This scaling is different from that inferred by Bostock *et al*.^29^ in the analysis of LFEs in Cascadia, where a much weaker scaling between seismic moment and corner frequency was inferred (*M*_0_ ∝ *f*_*c*_^−10^). It is worth to note that in that study LFEs were detected by a different method, i.e. a template-based matched filter method. Explanations other than classical shear rupture have been suggested to explain possibly these weaker scaling, such as simple forces acting in the direction of fluid transients in porous media^49^.

The probability, for our observations, of a scaling parameter smaller than −7 is less than $3\times {10}^{-4}$ (Fig. 2b). This probability keeps being small (0.06) even when the seismic moment domain is reduced to half a decade (Supplementary Fig. 8b). As such, an almost flat log *M*_0_ - log *f*_*c*_ scaling is very unlikely for the LFEs dataset analysed in this study, even over a small range of seismic moment.

Our result also differs from the scaling parameter of ~−1.5 that was reported for a limited number of VLFEs by Ide *et al*.^50^, and from the scaling parameters of ~ −2.0, −2.5 estimated by Ide^51^ and Ide *et al*.^52^ using a Brownian motion model for slow earthquakes. The latter values might however be consistent within a 2-sigma confidence interval with our observations but only when the seismic moment domain is reduced to one decade (Supplementary Fig. 8a).

While analysing the LFE component of the slow earthquakes family we found a different behaviour when compared to Ide *et al*.^24^, who retrieved an average scaling parameter of −1. It is worth to note here that a change of the scaling parameter from −3 to −1 has also been interpreted^25^ as a possible geometric effect associated to the growing of the rupture from 2-D to 1-D.

While a classical scaling parameter of −3.0 implies a constant stress drop – for a constant rupture speed – and a self-similar rupture process for the low-frequency earthquakes analysed in Nankai, a scaling of −3.5 suggest that the stress drop might weakly change with the rupture size. However, when exploring different values of the rupture velocity, we always found that stress drop variations remain smaller than one order of magnitude in the explored seismic moment range. A −3.5 scaling might also suggest variation of the rupture speed among the LFE sources, while the stress drop remains constant; in this case, the expected variation is much smaller than one order of magnitude^53^. At this stage, uncertainty in the estimations does not allow to discriminate between these different possible behaviours and a regular earthquake one. As such, in the remaining discussion we stick to the latter and simpler interpretation.

Although the scaling parameter of the LFEs looks similar to the one of the regular earthquakes, these events show on average a larger high-frequency spectral fall-off (3.0) than the one observed for regular earthquakes (2.0), indicating that LFEs are actually depleted in high-frequency radiation, when compared to regular events. This might be the signature of a smother arrest phase where the rupture does not brutally stop at barriers^54^, as it could be expected in a brittle-ductile transition environment.

Combination of the seismic moment and the rupture size allows estimation of the static stress drop^55^. The rupture size can be inferred from the corner frequency *f*_*c*_, when a kinematic model for the rupture is assumed together with a specific rupture velocity *v*_*R*_^56^.

**Figure 3:**
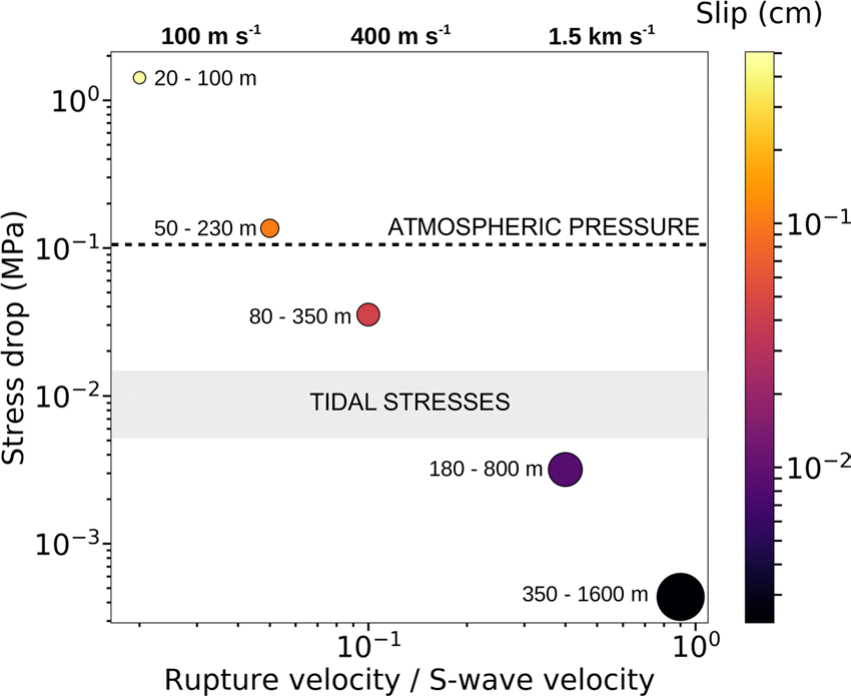
Stress drop, rupture dimensions and average slip as a function of the rupture velocity. The constant stress drop estimated using the scaling of Fig. 2 is shown (coloured circles) for a rupture velocity that varies from 0.02 *β* (S-wave velocity) to 0.9 *β* (see Methods; Supplementary Table 1). The minimum and maximum rupture dimensions of the LFEs are shown (circle labels), as are their average slips (circle colours). The sizes of the circles are scaled to the average rupture dimensions. Tidal stresses^58^ are shown (grey box). Reference values for the rupture velocity are given at the top of the Figure, assuming *β* = 3.7 km s^−1^.

The stress drop strongly depends on the rupture speed, and can increase by several orders of magnitude when *v*_*R*_ is reduced. Using the kinematic model of Sato and Hirasawa^56^ for a circular rupture, we analysed the dependence of the rupture size and stress drop of the LFE source on the rupture speed (see Methods; Supplementary Fig. 12). As shown in Fig. 3, when *v*_*R*_ decreases from 0.9*β* to 0.02*β*, where *β* is the ambient shear-wave speed, the stress drop increases from 10^3^ to 10^6^ Pa.

A very low stress drop in the kilopascal range is only derived under the assumption of a fast rupture speed (Fig. 3), as observed for regular earthquakes, and is consistent with previous studies^29,40,57,58^, together with an average slip of the order of tens of micrometres over sub-kilometre rupture sizes. Stress drop of the order of 10^6^ Pa is derived when assuming a value of 2% of *β* as the lower end member for *v*_*R*_, together with an average slip of the order of 10 cm over rupture sizes that vary from 20 m to 100 m. The source scaling between seismic moment and corner frequency cannot constrain the size, the slip and the stress drop of the LFE sources, as long as independent estimations of the rupture velocity or the rupture size are not available.

At this stage, such an interpretation of the results of this study raises challenging questions.

A rupture speed close to the asymptotic limit leads to a stress drop lower than expected for tremor modulation by tides and surface waves of large teleseismic earthquakes^57,59^. It is also difficult to reconcile a micrometre scale for the slip and a wide kilometre-scale rupture occurring along a heterogeneous plate interface.

On the other hand, extremely low rupture speeds are associated with a stress drop up to 10^6^ Pa. which is hard to reconcile with the observation that the activity of LFEs can be strongly modulated by much smaller stress changes^57,59^. Moreover, slip and stress drop in this parameter domain lead to an energy budget of the same order as for regular earthquakes. Thus, a very different and highly dissipative rupture dynamics is required to limit the effective rupture speed at such a small fraction of the shear-wave speed.

In conclusion, the central parameter domain of Fig. 3 seems the most likely. A rupture velocity smaller than 0.4*β* is compatible with previous modelling of apparent LFE source time functions^60^. For velocities down to ~0.05*β* the related stress drop scale (1–100 kPa) remains consistent with a modulation of the LFEs activity by tides and teleseisms while the averaged slip varies from 0.1 mm to 1.0 mm over averaged rupture sizes from 400 m to 100 m. Assuming for example *v*_*R*_ = 0.1*β* and *β* = 3.7 km s^−1^, the stress drop varies from 22 kPa (log M_0_ = 10.4, f_c_ = 4.6 Hz) to 40 kPa (log M_0_ = 12.4, f_c_ = 1.2 Hz).

## Methods

### Source parameters estimation

We used a probabilistic method^39^ based on the conjunction of states of information between the data and the model to retrieve the LFE source parameters from the joint PDF expected over the model space when both model and data uncertainties are assumed to be normally distributed. We model the S-wave far-field amplitude displacement spectrum of Eq. (3),

3 $$\tilde{u}(f)=\tilde{S}(f)\tilde{G}(f),$$

where *f* is the frequency, $\tilde{S}(f)$ is the modulus of the Fourier transform of the source–time function, and $\tilde{G}(f)$ is the modulus of the Fourier transform of the Green’s propagator.

The source spectrum is modelled assuming a generalised Brune’s spectral model^37^, as in Eq. (4):

4 $$\tilde{S}({M}_{0},{f}_{c},\gamma ;f)={M}_{0}/(1+{(f/{f}_{c})}^{\gamma }).$$

The model space is defined by three source parameters: the seismic moment, *M*_0_, which is related to the energy released by the source; the corner frequency, *f*_*c*_, which is a proxy for the rupture length; and the high-frequency fall-off exponent γ.

The Green’s propagator $\tilde{G}(f)$ is assumed to have a frequency-independent attenuation quality factor *Q*^61^, which was fixed at 300, as provided in the literature^38^.

### Signal processing

We applied the following methodology to each single station S-wave displacement spectrum. The S-wave arrival times *T*_*S*_ are theoretically obtained from the one-dimensional layered velocity model of Kubo *et al*.^38^. A 4 s S-wave time window was selected (Eq. (5)), together with a noise time-window of the same duration (Eq. (6)):

5 $$\Delta {T}_{S}=[{T}_{S}-1s,{T}_{S}+3s],$$

6 $$\Delta {T}_{N}=[{T}_{0}-4s,{T}_{0}],$$

where *T*_0_ is the origin time of the event.

The raw signal was processed to remove the instrumental response together with both the constant and linear trends; Hann-function tapering was applied to the first and last 5% of the signal. The signal and noise amplitude spectra were derived by applying fast Fourier transform to the pre-processed signal and noise time windows, respectively. Finally, each spectrum was smoothed in a logarithmic scale using a five-point moving average filter.

For each LFE and each station, the geometrical mean of the smoothed spectra of the two horizontal components was inverted^62^.

### Single-station solution

The LFE signals are characterised by very low signal-to-noise ratios, which are usually a little larger than 1 (Supplementary Fig. 2a). Even when the S-wave train emerges in the time domain, its amplitude is of the same order of magnitude as the noise amplitude, which can affect the spectral shape. Nevertheless, we can observe a region in the frequency domain around the LFE corner frequency where the S-wave spectrum is actually larger than the noise spectrum. This sub-domain is usually large enough to resolve the low-frequency flat level and the high-frequency fall-off decay in the S-wave spectrum (Supplementary Fig. 2b).

The spectral modelling is restricted to the frequency sub-domain where the signal amplitude is at least 1.25-fold the noise. In the example in Fig. 2b, this region corresponds to the interval [0.8–5.2] Hz, which is indicated by the black horizontal arrows. We invert the displacement spectrum in the selected frequency band to retrieve the joint PDF for the source parameters, with the estimation of the expected value and related uncertainty for each parameter as the mean and the standard deviation of the corresponding marginal PDF (Supplementary Fig. 2c). In Supplementary Fig. 2b, we show an example of the theoretical spectrum, as calculated with the estimated source parameters, and superimposed on the observed spectrum. We also show in Supplementary Fig. 2d the 2-D marginal PDFs for each couple of parameters.

### Quality selection criteria

We automatically discard noisy data for which the selected frequency sub-domain where the S-wave spectrum is above the noise spectrum is reduced to less than 10 points (90% of rejections). Moreover, for some records, the signal-to-noise ratio can be too low, which leads to an unconstrained PDF in terms of at least one parameter. This allows automatic detection and discarding of these unconstrained solutions^39^ (10% of rejections).

Application of these two criteria resulted in rejection of about 75% of the LFE events in the catalogue (Supplementary Fig. 1).

### Event solution

Source parameters for an event are obtained as the weighted means of single-station estimations, where the weights are the inverse of the variances, and their uncertainties are given by the standard errors^39^.

### Corner frequency–seismic moment scaling

We bin the event solutions in the seismic moment domain (Fig. 2) to estimate the scaling coefficient between the corner frequency and the seismic moment. The size of each bin was 0.03. The seismic moment estimation corresponds to the centre of the bin, while the corner frequency is the weighted mean of the event solutions belonging to the bin. The variability of the corner frequency measurements in the bin is represented by the weighted standard deviation shown in Eq. (7);

7 $${\bar{\sigma }}_{W}=\sqrt{\mathop{\sum }\limits_{i=1}^{N}[{({f}_{{c}_{i}}-\overline{{f}_{c}})}^{2}/{\sigma }_{{f}_{{c}_{i}}}^{2}]/\mathop{\sum }\limits_{i=1}^{N}{\sigma }_{{f}_{{c}_{i}}}^{-2}},$$

where N is the total number of event solutions in the bin (Fig. 2).

In the spectral inversion, we do not consider site effects^39^. The average of the corner frequencies in each bin comes from a large number of stations and mitigates possible single-station site effects, if any.

## Probability density function of the scaling parameter

We estimated the PDF of the scaling parameter (Fig. 2a, Supplementary Fig. 8) through a Bootstrap method. We randomly extract a single value of *f*_*c*_ per seismic moment bin from a normal distribution parameterised by the mean and variance of the bin (Fig. 2a). This provides a new collection of corner frequencies as a function of the seismic moment. For this set of couples (*M*_0_, *f*_*c*_), the scaling parameter is estimated, as discussed in the main text. This extraction procedure is repeated 100,000 times to obtain a good approximation of the PDF of the scaling parameter from the normalised histogram of the estimated scaling parameters.

We estimated the PDF for 3 different seismic moment domains, to assess the robustness of the results. First, we used all the available observations (Fig. 2a); then, we reduced the domain to a decade, selecting the 75% of the observations (Supplementary Fig. 8a). Finally, we reduced the seismic moment domain to half a decade, selecting the 44% of the observations (Supplementary Fig. 8b).

## Dependence of the stress drop on the rupture velocity

The corner frequency *f*_*c*_ is a proxy for the rupture size *r*. Assuming a kinematic model for a self-similar circular rupture, that expands at a constant rupture velocity *v*_*R*_, expressed as a fraction of the S-wave velocity *β*, the corner frequency is a function of the shear-wave speed and the source size *r, f*_*c*_ = *k β*/*r*, where *k* is a constant that depends on the rupture speed^56,63,64^.

We analyse the dependence of the rupture size, and therefore of the static stress drop $\varDelta \sigma \propto {M}_{0}/{r}^{3}$^55,65^, on the rupture velocity. For the sake of simplicity, we used the kinematic circular crack model of Sato and Hirasawa^56^ to estimate the *k* coefficient for different *v*_*R*_. This model has an analytical representation for the far-field displacement spectrum that allows computing the synthetic spectra for different rupture speeds and different take-off angles. From the synthetic spectra, we estimate the source parameters (seismic moment, corner frequency and high-frequency decay exponent) using the probabilistic method of Supino *et al*.^39^.

We estimate the *k* coefficient^56^, averaging the different corner frequencies obtained for a take-off angle *θ* from 0° to 90°, with a discretisation step of 5°, to remove the expected directivity effects (Supplementary Fig. 12a). Sato and Hirasawa^56^ provided *k*-values for *v*_*R*_ ranging from 0.5 *ß* (k = 0.25) to 0.9 *ß* (k = 0.32). We have obtained the same coefficient values, and extended those to slower rupture velocities. We derived *k = *0.214 for *v*_*R*_ = 0.4 *ß*, *k = *0.096 for *v*_*R*_ = 0.1 *ß*, *k* = 0.061 for *v*_*R*_ = 0.05 *ß*, and *k* = 0.028 for *v*_*R*_ = 0.02 *ß*. The estimated source radius decreases as *v*_*R*_ decreases (Fig. 3), leading to an increase of the stress drop $\Delta \sigma \propto {M}_{0}/{r}^{3}$.

It is worth to note that this simple kinematic rupture model does not fully capture the high frequency behaviour of the LFE sources, for which a fall-off exponent around 3 is inferred in this study.

The radiated high frequencies in the seismograms are dominated by the stopping phases, which contain the directivity factors, emitted from the closest and the farthest points of the rupture, and are inherent to the prescribed brutal arrest of the circular rupture at its final size.

As such the Sato and Hirasawa^56^ model predicts a high frequency fall-off exponent around 2 for fast constant rupture velocities, while the exponent is 1 at the limit value *v*_*R*_ = 0. The latter is not physically admissible since energy would then diverge. Therefore, for all the rupture speeds considered in this analysis, we checked (Supplementary Fig. 12b) that the predicted high-frequency falloff always decays with an exponent larger than 1.5, which is the limit value above that the energy converges.

More complex models, with additional parameters, have been proposed to explain a high-frequency spectral fall-off of 3.0, such as models where the rupture does not stop brutally at barriers but decelerates more smoothly^54^. Circular or elliptic ruptures with hypocentre not at the centre of the source show azimuthal domains in which the spectral fall-off is larger than 3.0^66^. However, for all these models the change in the *k* values leads only to a change in stress drop smaller than one order of magnitude. The same is true when considering self-similar dynamic source models, like the Madariaga circular crack model^63^ (where k = 0.21) or the Kaneko and Shearer frictional circular rupture model^64^ (k = 0.26).

## Supplementary information

Supplementary information.

## Data Availability

The seismological time series used for this analysis are available from the National Institute for Earth Science and Disaster Prevention (http://www.hinet.bosai.go.jp). The catalogue of the LFE locations and source parameters is provided at the following URL (10.7910/DVN/HCWJUI). The software used for detection and location is available from Git-Hub (http://backtrackbb.github.io). The software used for the source parameter computation is available on request, contacting the corresponding author. The JMA unified earthquake catalogue is provided at the following URL (https://hinetwww11.bosai.go.jp/auth/?LANG=en).
