# Supplementary material for: Self-similarity of low-frequency earthquakes
Source: Sci Rep. 2020 Apr 16;10:6523. doi: 10.1038/s41598-020-63584-6 (PMC7162910; doi:10.1038/s41598-020-63584-6)
Supplement: Supplementary file 1 — Supplementary information. [file 41598_2020_63584_MOESM1_ESM.docx]

**Supplementary information for:**

**Self-similarity of low-frequency earthquakes**

M. Supino^1,2^*, N. Poiata^1,3^, G. Festa^2^, J.P. Vilotte^1^, C. Satriano^1^ and K. Obara^4^

^1^Université de Paris, Institut de physique du globe de Paris, CNRS, F-75005 Paris, France.

^2^Dipartimento di Fisica ‘Ettore Pancini’, Università di Napoli Federico II, I-80126, Napoli, Italy.

^3^National Institute for Earth Physics, 12 Călugăreni, Măgurele, 077125 Ilfov, Romania.

^4^Earthquake Research Institute, University of Tokyo, Bunkyo, Tokyo 113-0032, Japan.

| ***A* (slope)** | **1/*A*** | ***B* (intercept)** |
| --- | --- | --- |
| -0.287 ± 0.008 | -3.5 ± 0.1 | 3.65 ± 0.09 |
| -1/3 (fixed) | -3.0 | 4.179 ± 0.005 |

**Table S1.** Best fit parameters of the linear regression log *f_c_* = *A* log *M_0_* + *B*, using the bin-averaged corner frequencies (Fig. 2). The intercept *B* is also estimated fixing the slope to the value -1/3; this estimate is used to compute the values of stress drop shown in Figure 3.

**
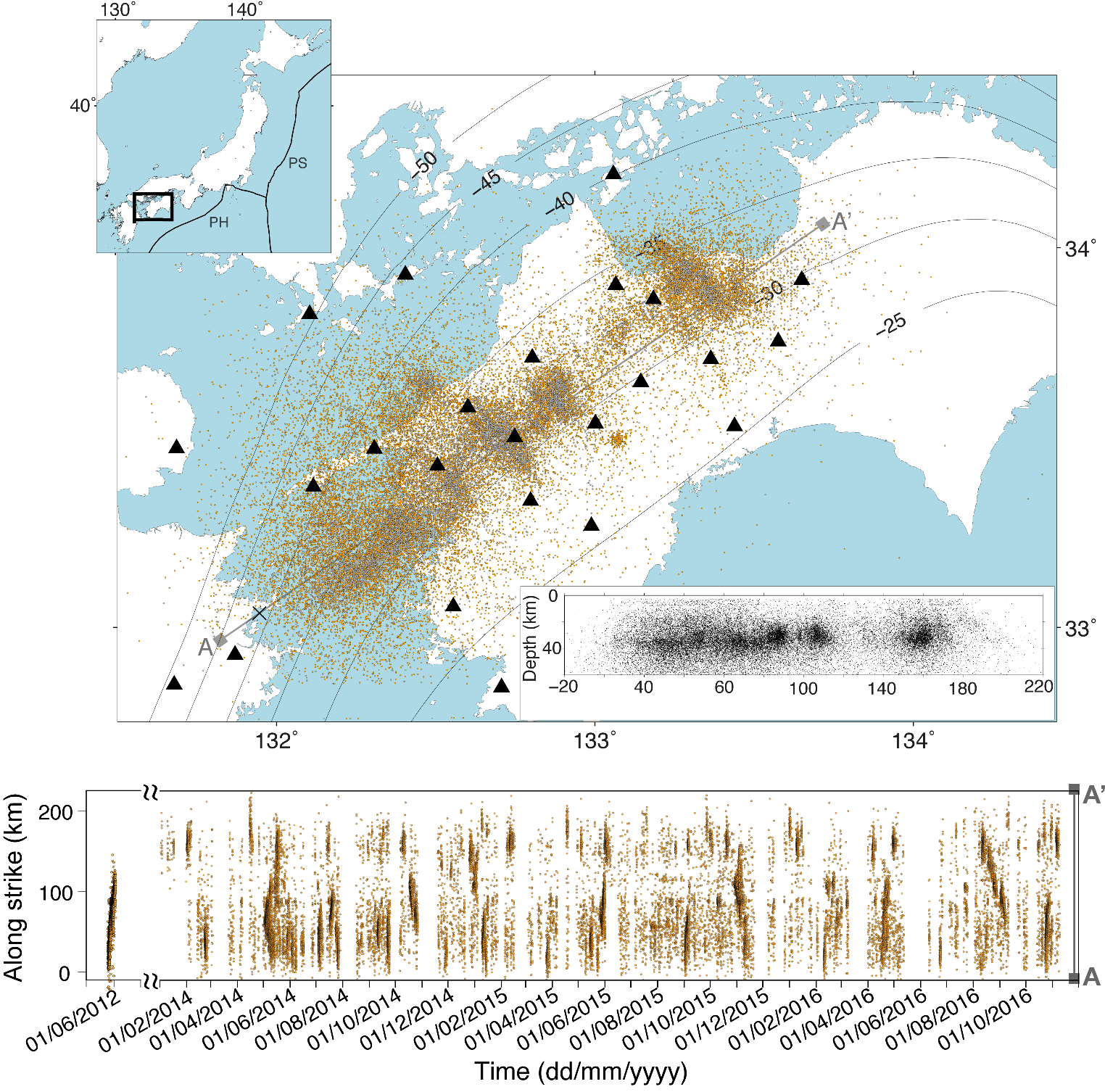
**

**a**

**b**

**Figure S1. Distribution of detected low-frequency earthquakes. a**, Map of the locations of the detected low-frequency earthquakes (brown circles) and the Hi-net stations (triangles). The events for which we estimated a source parameters solution are shown (grey circles) (see Methods). Inset, top: The geographic and tectonic settings of the western Shikoku area. Inset, bottom: The depth cross-section of events projected along the strike of N 40º E (A-A’). **b**, Space–time plot of the events. Colours as in (a).

**
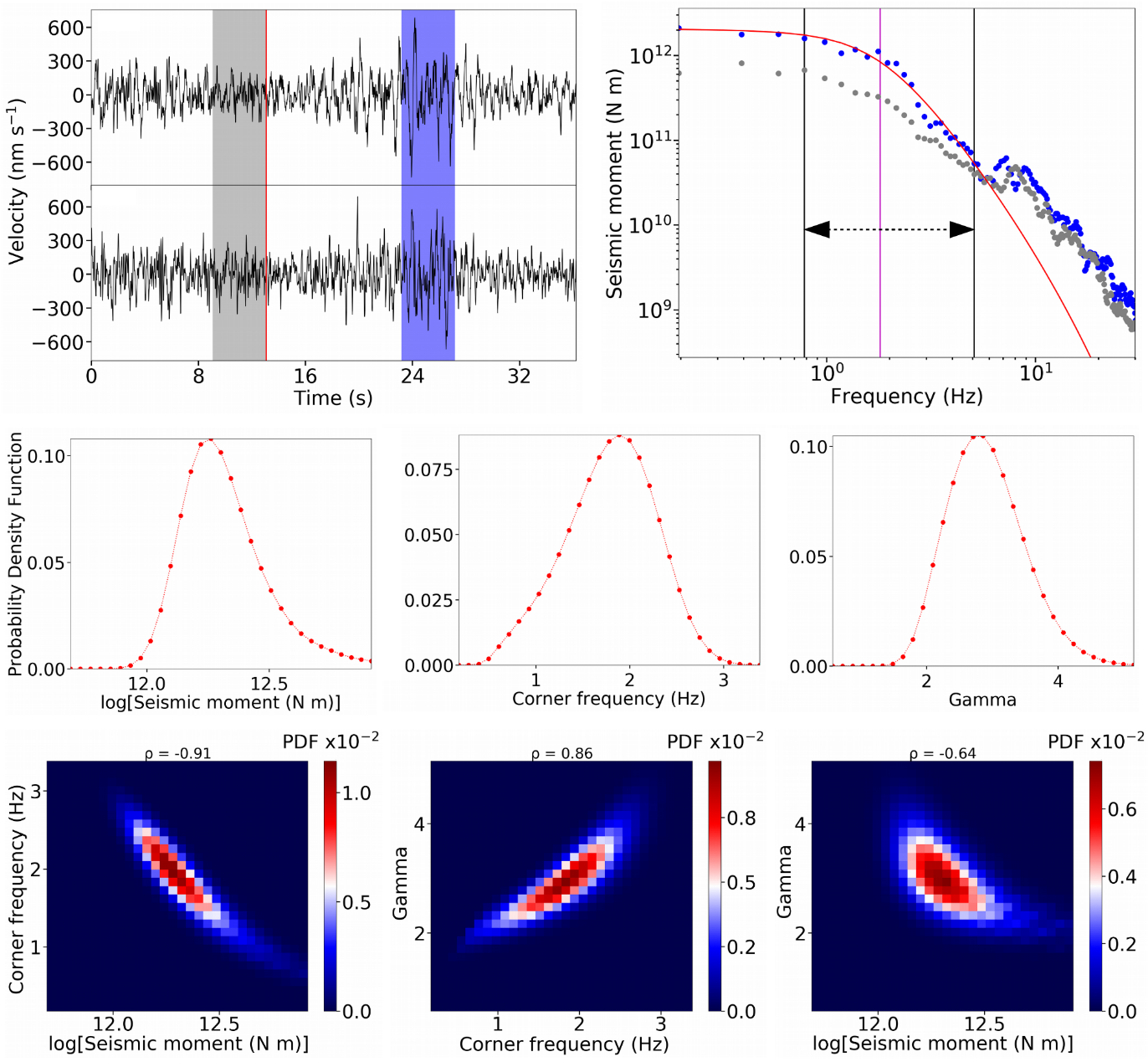
**

**d**

**a**

**b**

**c**

**Figure S2. Source parameter solutions for single-station observation.** **a**, Selected S-wave signal (blue box) and noise (grey box). The origin time of the event is shown (red bar). **b**, Displacement amplitude spectrum of the unfiltered S-wave signal (blue circles), the noise (grey circles), and the best-fit solution (red curve). The black arrows show the frequency domain selected for the inversion (see Methods). The vertical line shows the estimated corner frequency (magenta). **c**, Marginal probability density functions of the source parameters log *M_0_*, *f_c_* and *γ*. **d**, 2-D marginal probability density functions of the source parameters; correlation coefficients are at the top of each heatmap. Event-ID 20140505_2358H, Hi-net station N.UWAH.

**
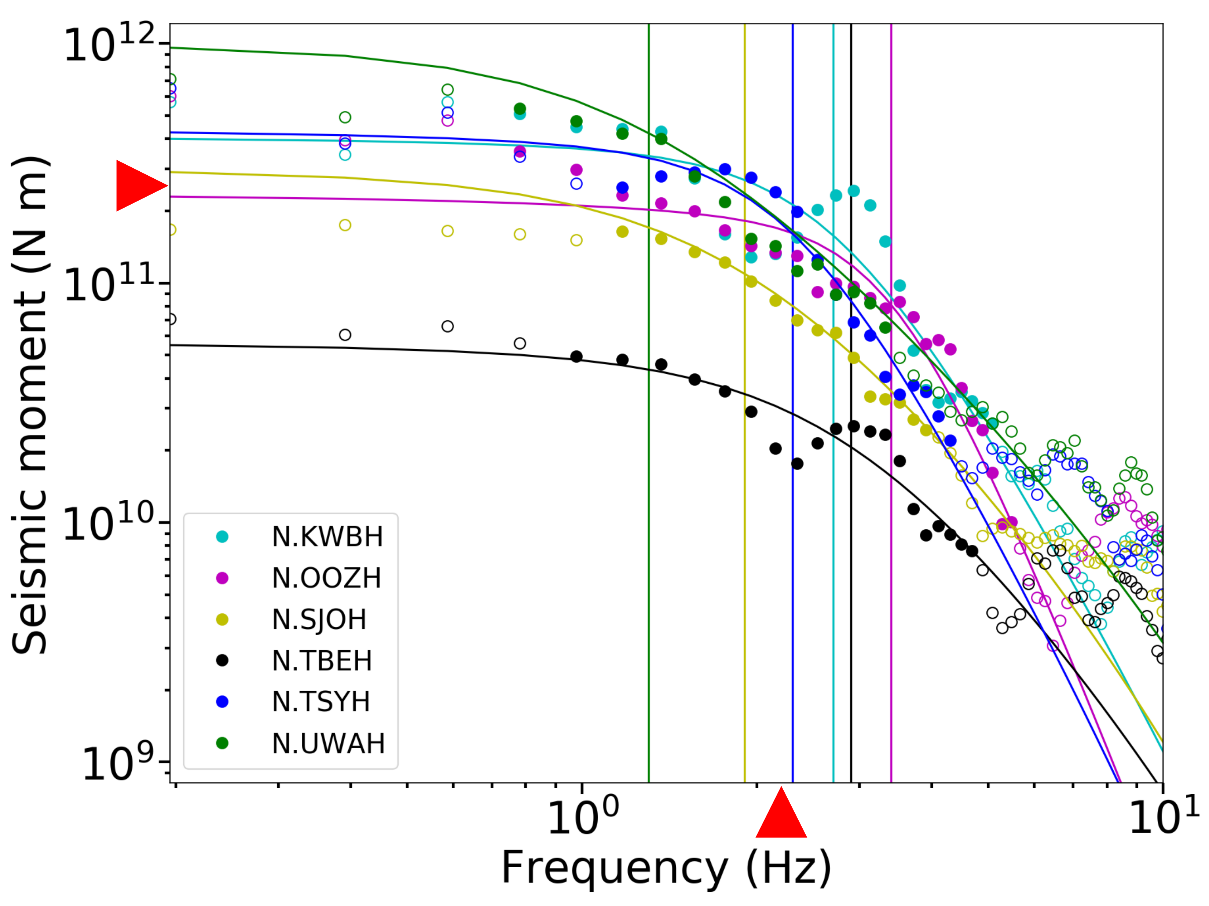
**

**Figure S3. Seismic moment and corner frequency variability for single-event solutions**. The single-station displacement spectra for which source parameters solutions were retrieved are shown (discrete curves), along with the best-fit solutions (continuous curves) and corner frequency estimates (vertical bars), the frequencies not selected for the inversion due to low signal-to-noise ratio (empty circles) (see Methods), and the corner frequency and seismic moment estimates for the event (red arrowheads). Event-ID 20150530_1157H, Hi-net stations, see Key.

**a**

**b**

**c**


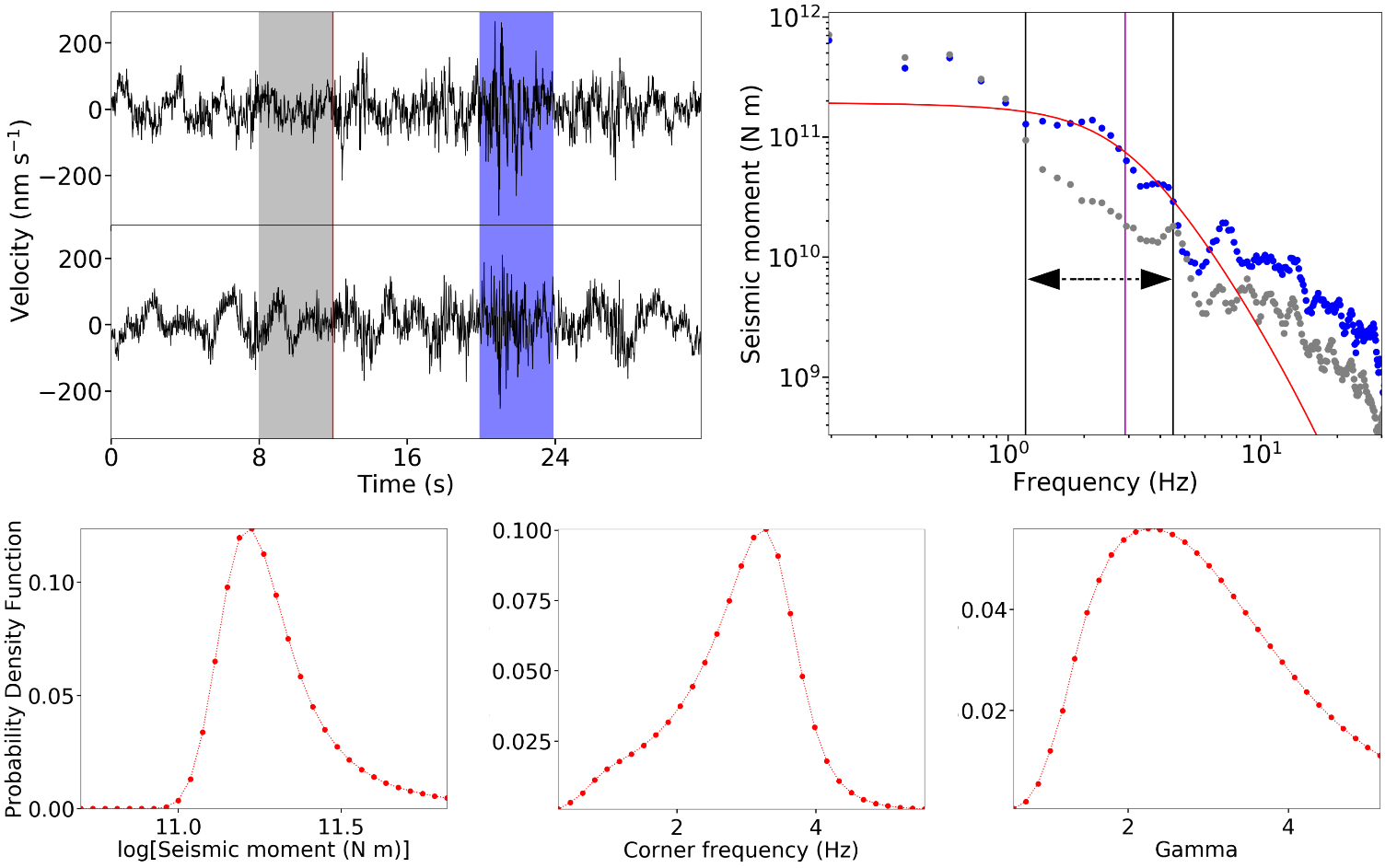


**Figure S4. Source parameter solutions for single-station observation with log *M_0_* = 11.3.** **a**-**c,** As for Supplementary Figure 2. Event-ID 20150212_0123Q, Hi-net station N.GHKH.

**a**

**b**

**c**


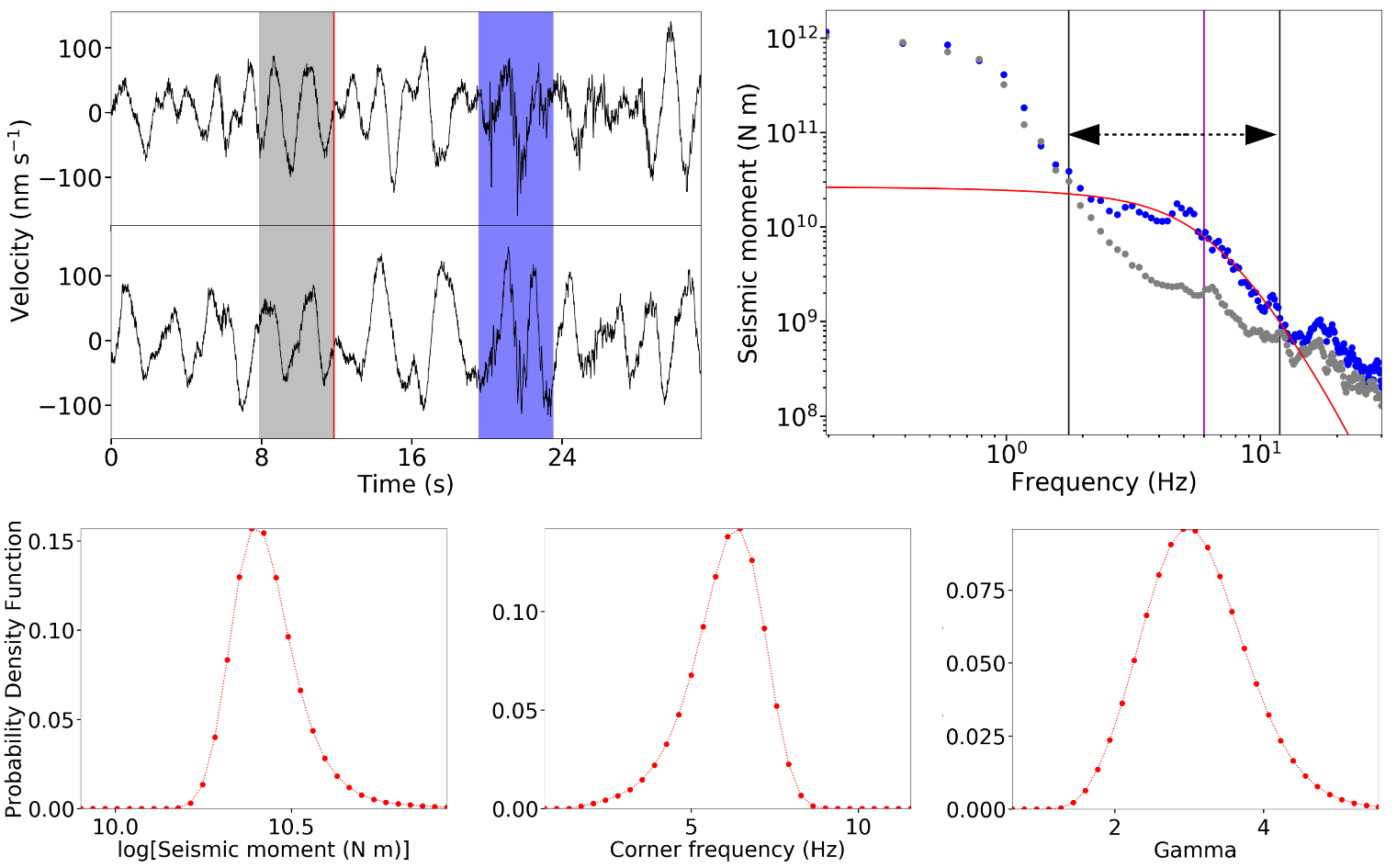


**Figure S5. Source parameter solutions for single-station observation with log *M_0_* = 10.4.** **a**-**c,** As for Supplementary Figure 2. Event-ID 20151108_0620B, Hi-net station N.KWBH.


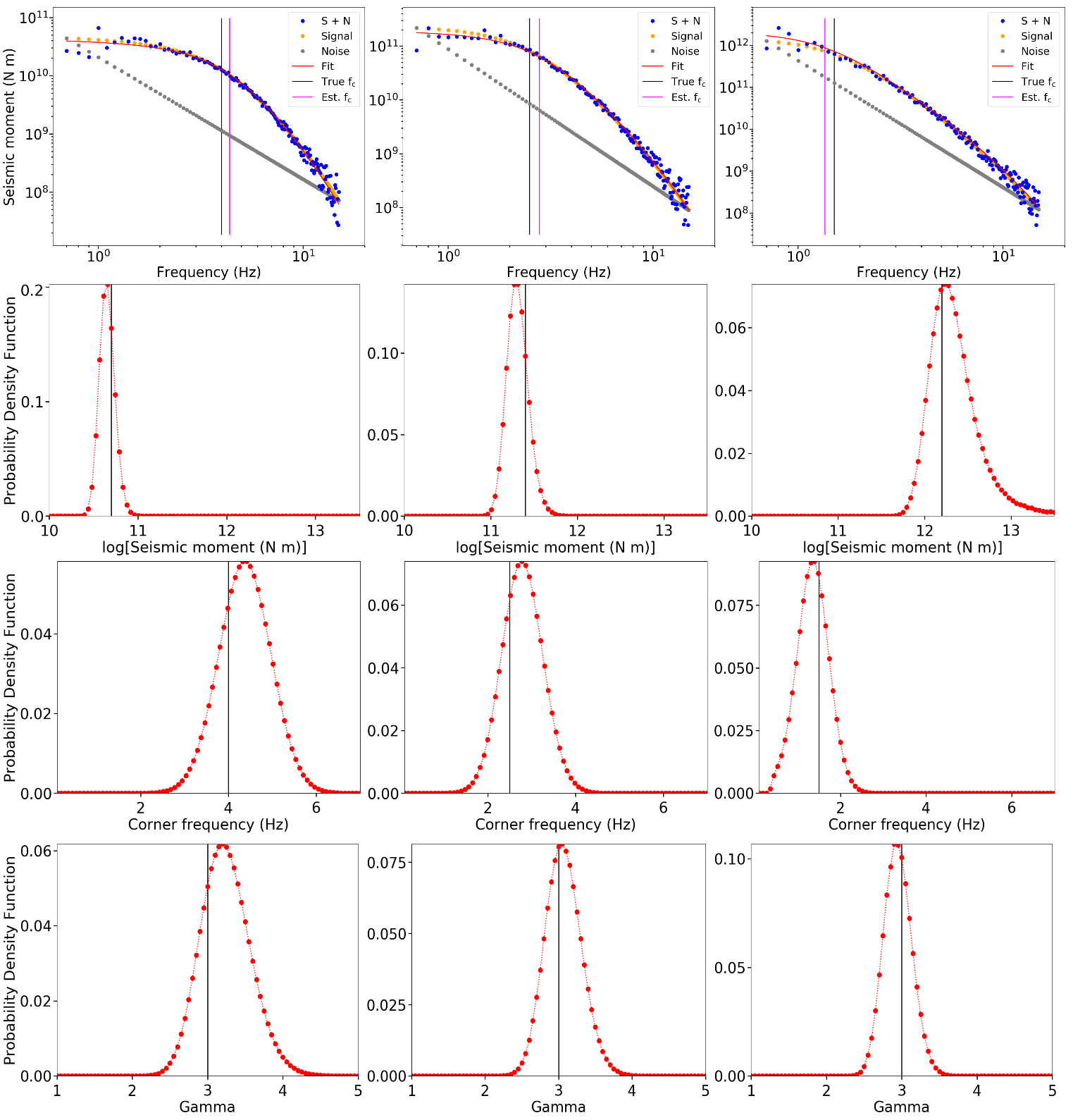


**c**

**a**

**b**

**Figure S6. Synthetic tests for the explored seismic moment domain.** Source parameter PDFs estimated from the inversion of synthetic spectra given by the addition of theoretical (Brune) source spectrum and noise spectrum, imposing a signal-to-noise ratio > 1.25, for different log M0 – fc couples. **a**, Theoretical source parameters: log *M_0_* = 10.7, *f_c_* = 4 Hz, *γ* = 3. Upper panel: the inverted synthetic spectrum is shown (blue circles), along with the source spectrum (orange circles), the noise spectrum (grey circles), the best-fit solution (red curve), the true corner frequency (black vertical bar) and the estimated corner frequency (magenta vertical bar). Lower panels: the marginal PDFs of log *M_0_*, *f_c_* and *γ* are shown (red circles), along with the true values of the source parameters (black vertical lines). **b**, as for (a) with log *M_0_* = 11.4, *f_c_* = 2.5 Hz. **c**, as for (a) with log *M_0_* = 12.2, *f_c_* = 1.5 Hz.

**
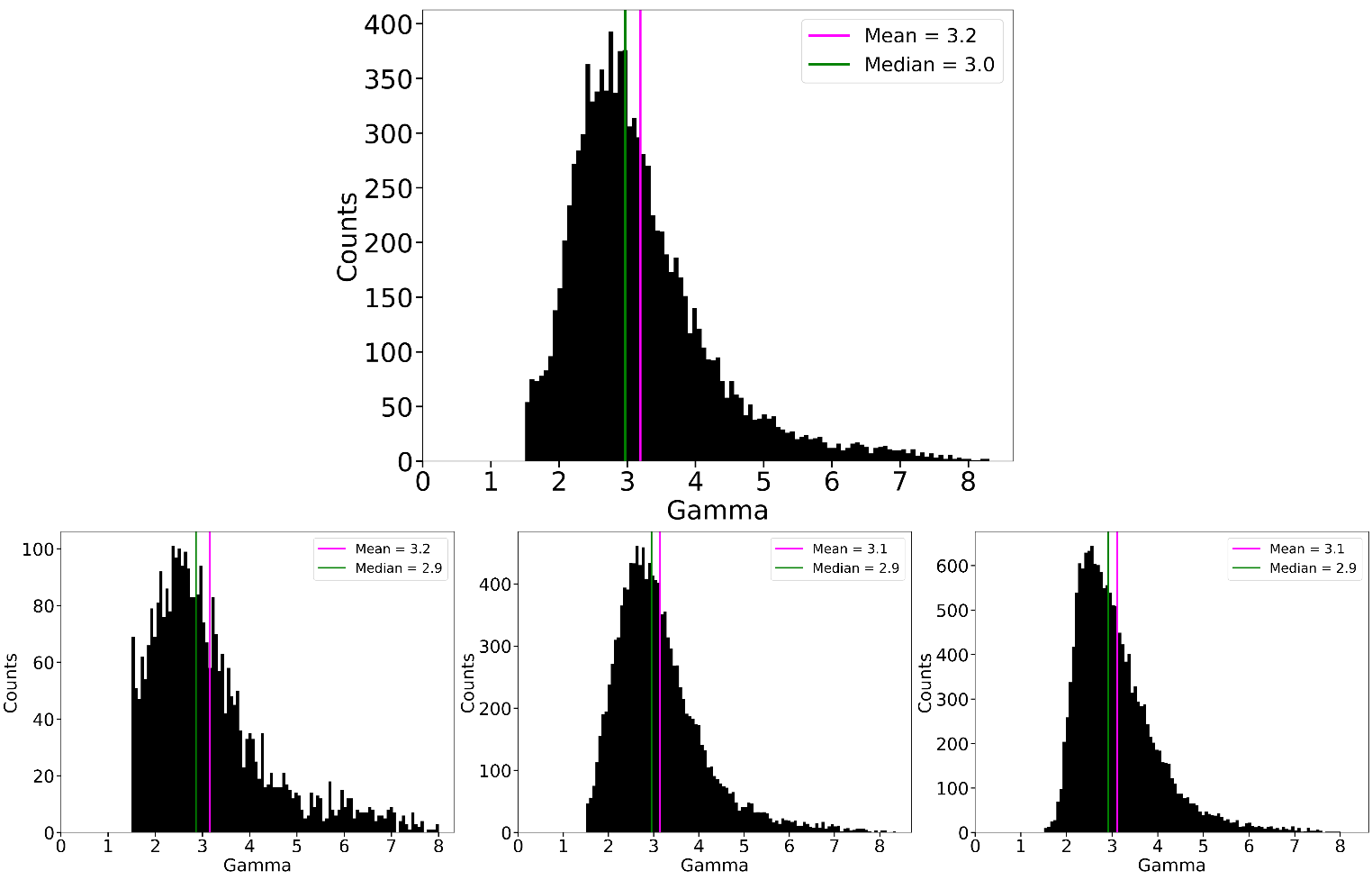
**

**a**

**b**

**c**

**d**

**Figure S7. Histogram of the high-frequency decay exponents estimated assuming different attenuation factors. a**, Histogram of the gamma estimates when Q = 300. The mean of the distribution (magenta line) is 3.2, the median (green line) is 3.0. **b**, As for (a), with Q =100; the mean is 3.2, the median is 2.9. **c**, As for (a), with Q = 500; the mean is 3.1, the median is 2.9. **d**, As for (a), with Q(f) = Q_0_ f ^ε^, where log (Q_0_)^-1^ = -2.5 and ε = 0.5; the mean is 3.1, the median is 2.9.

**b**

**a**

**
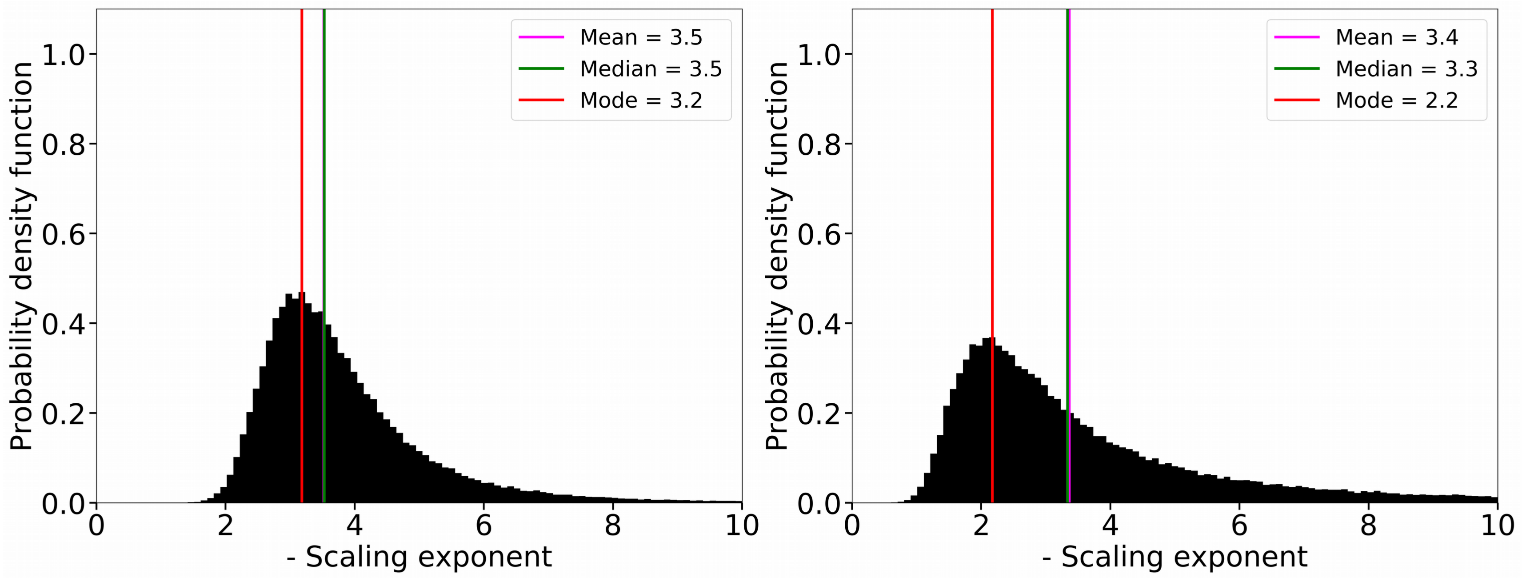
**

**Figure S8. PDF of the scaling exponent.** Probability density functions of the scaling exponent estimated with a bootstrap method performed with 100,000 random extractions (see Methods). **a**, Bootstrap performed using half of the seismic moment domain shown in Figure 2 (log *M_0_* = 11.0 – 12.0). **b**, Seismic moment domain used for bootstrap test is reduced to half a decade (log *M_0_* = 11.0 – 11.5).


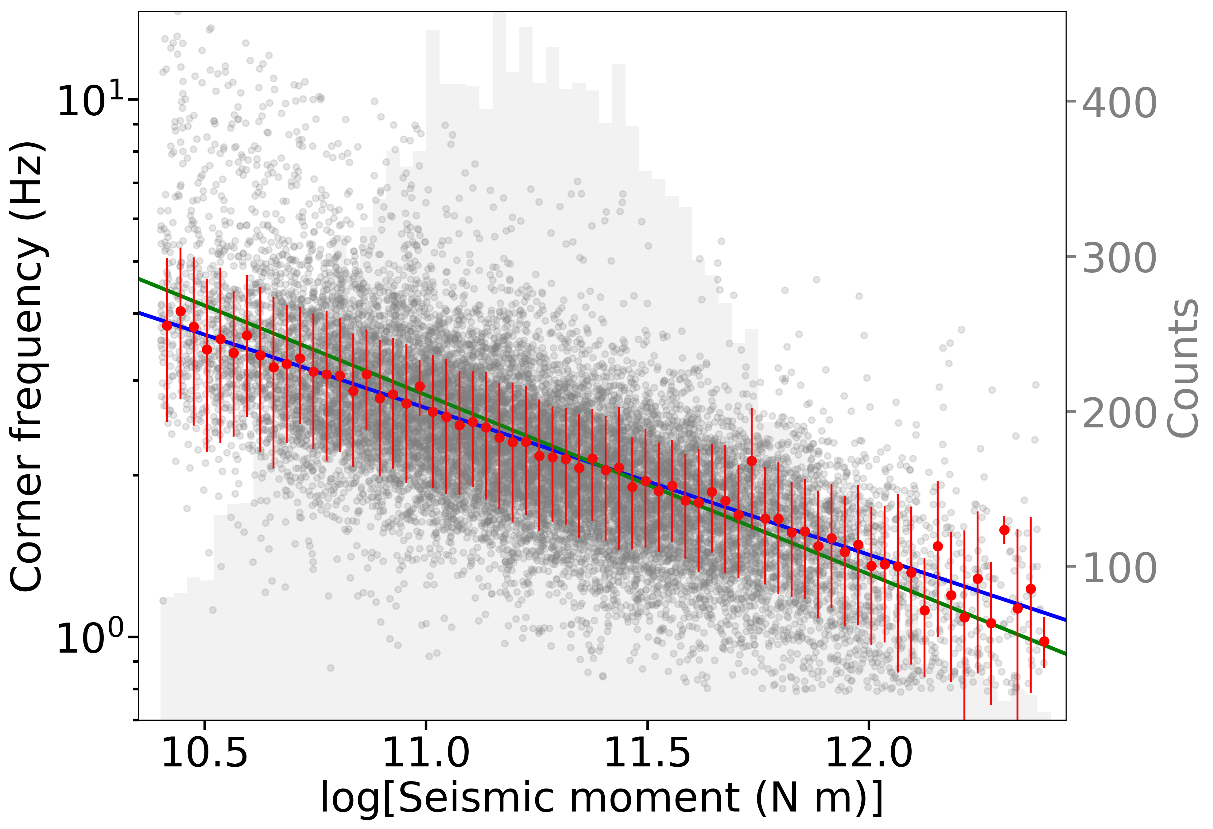


**Figure S9. Scaling of the corner frequency with the seismic moment, assuming a frequency-dependent Q.** As for Figure 2. The best-fit curve (blue line) of the averaged estimates (red points) has a scaling exponent of -3.7. The green line represents the scaling of -3.

**a**


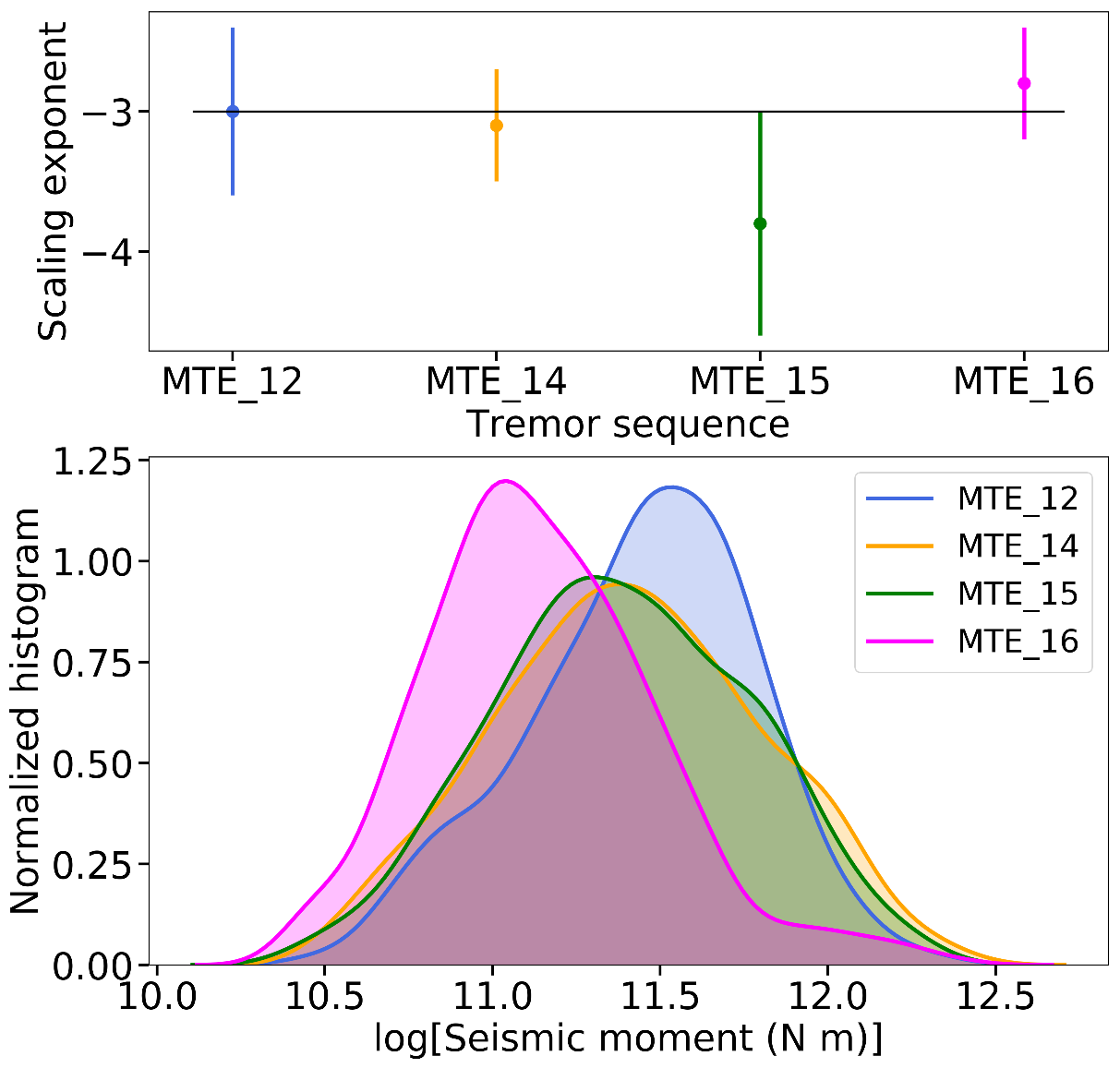


**b**

**Figure S10. Scaling exponents for low-frequency earthquake clusters. a**, The scaling exponent is estimated as shown in Figure 2, for different clusters of low-frequency earthquakes. MTE_12, MTE_14, MTE_15 and MTE_16 refer to the major tremor sequences of 2012, 2014, 2015 and 2016, respectively. Error bars: 2-σ standard error. **b**, Normalised histograms showing the explored seismic moment domain for each cluster, with colours as for the top panel. The distributions are similar between the clusters.


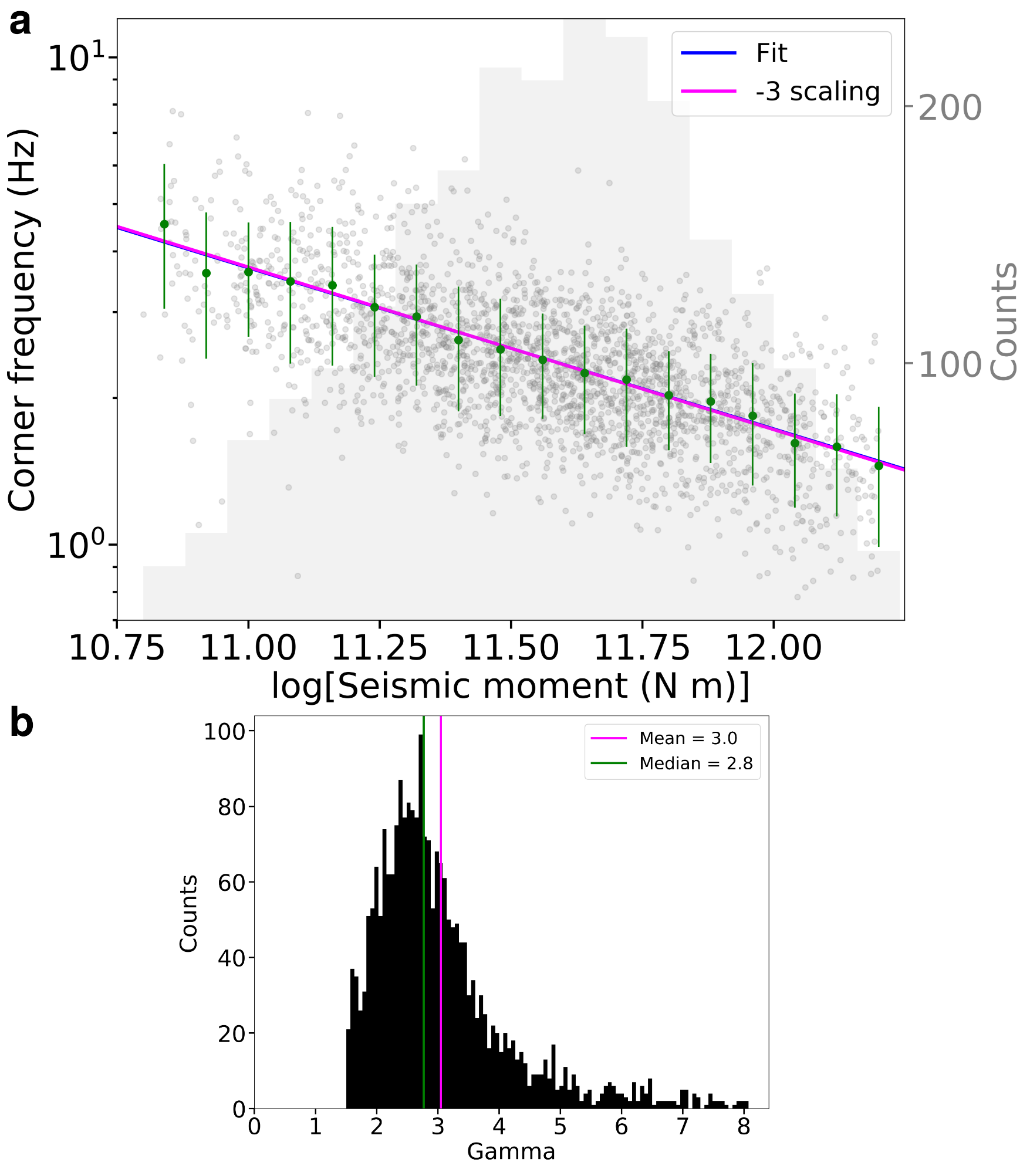


**Figure S11. Scaling of the corner frequency with the seismic moment for the JMA catalogue, and corresponding gamma estimates.** **a**, The corner frequency and seismic moment estimates for each LFE are shown (grey points). The weighted averages of the corner frequencies for the selected seismic moment bins (bin-size, 0.08) are shown (green points), along with the weighted standard deviations per bin (green bars). The best-fit curve (blue line) of the averaged estimates (green points) has a scaling parameter of -3.02. The histogram in the background (grey shading) shows the number of events in each bin. The magenta line represents the scaling of -3. **b**, Histogram of the high-frequency decay exponents. The mean of the distribution (magenta line) is 2.8, the median (green line) is 3.0.


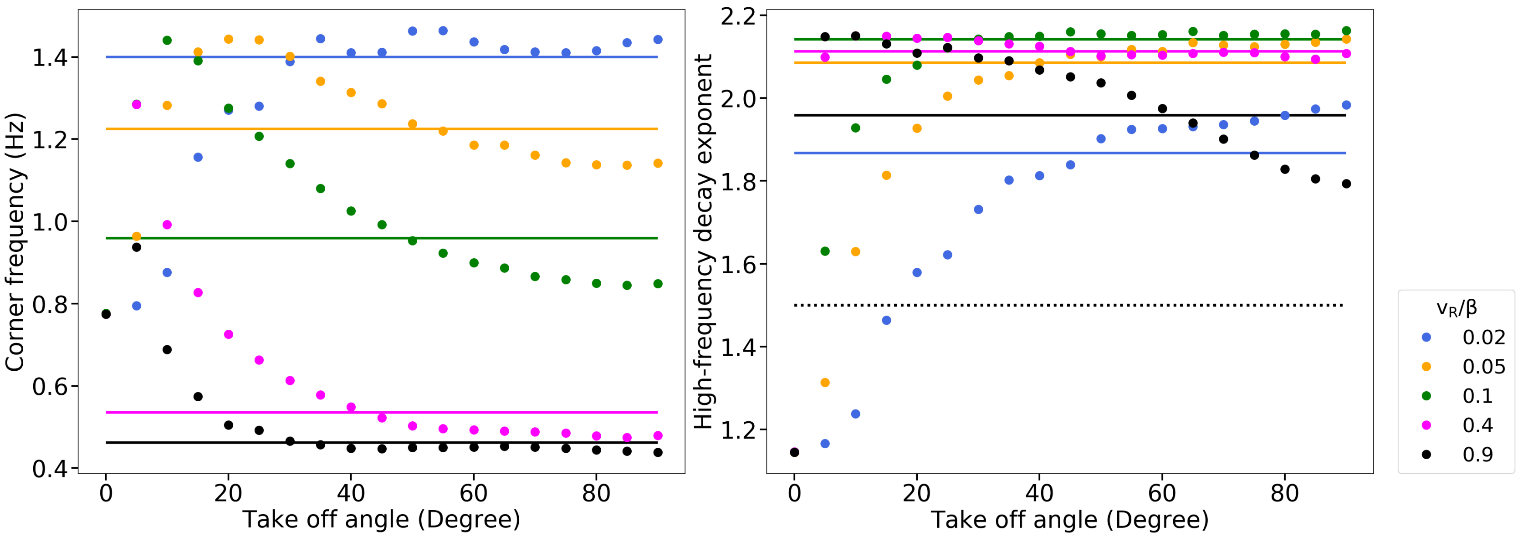


**a**

**b**

**Figure S12. Corner frequencies and high-frequency decay exponents as a function of take-off angles for different rupture velocities.** **a**, Corner frequencies are estimated from synthetic spectra generated using the Sato and Hirasawa model^46^, for take-off angles from 0° to 90°, with discretisation step of 5° (see Methods). **b**, High-frequency decay exponents are estimated as for (a). Colours referred to the different *v_R_*/*ß* used to generate the spectra. *v_R_*, rupture velocity; *ß*, shear-wave velocity, *v_R_*/*ß*, see Key.
